# Midgut Volvulus Secondary to Intestinal Malrotation in an 11-Year-Old Child With Preserved Bowel Viability: A Rare Delayed Presentation

**DOI:** 10.7759/cureus.112427

**Published:** 2026-07-10

**Authors:** Abhishek Mohanty, Ali Z Anwar, Sunil Kumar

**Affiliations:** 1 General Surgery, Shahid Nirmal Mahato Medical College and Hospital, Dhanbad, IND; 2 Surgical Oncology, Kidwai Memorial Institute of Oncology, Bangalore, IND

**Keywords:** acute abdomen in children, bilious vomiting, delayed presentation, intestinal malrotation, ladd’s procedure, midgut volvulus, pediatric intestinal obstruction, pediatric surgery, whirlpool sign

## Abstract

Midgut volvulus is a life-threatening surgical emergency that most commonly presents during infancy, whereas delayed presentation in older children is uncommon and can be diagnostically challenging. An 11-year-old boy presented with acute abdominal pain, bilious vomiting, abdominal distension, and obstipation of two days’ duration after four prior episodes of subacute intestinal obstruction over three years that had been managed conservatively. Contrast-enhanced computed tomography of the abdomen demonstrated the classical whirlpool sign, suggestive of midgut volvulus secondary to intestinal malrotation. Emergency exploratory laparotomy confirmed incomplete intestinal malrotation with a 180-degree clockwise midgut volvulus around a narrow mesenteric base. The bowel was congested but fully viable, without ischemia or necrosis. The patient underwent Ladd’s procedure with counterclockwise derotation, division of Ladd’s bands, widening of the mesenteric base, and bowel repositioning. Caecopexy and sigmoidopexy were also performed because of marked intraoperative mobility of the caecum and redundant sigmoid colon. Recovery was uneventful, and the patient was discharged on postoperative day 7. This case highlights the need to consider malrotation with volvulus beyond infancy in children with recurrent bilious vomiting or episodic intestinal obstruction, because early imaging and timely surgery can preserve bowel viability.

## Introduction

Intestinal malrotation is a congenital anomaly resulting from abnormal rotation and fixation of the midgut during embryological development. Although the condition may remain asymptomatic, it predisposes patients to midgut volvulus, a surgical emergency caused by twisting of the bowel around a narrow mesenteric pedicle. Intestinal malrotation with midgut volvulus most commonly presents during infancy. However, a substantial proportion of patients present during the neonatal period; nearly 75-90% of symptomatic cases become evident within the first year of life [1]. Presentation beyond infancy is uncommon and frequently associated with delayed or missed diagnosis because symptoms may be intermittent, nonspecific, and recurrent over several years. Older children may present with recurrent abdominal pain, episodic bilious vomiting, or features of subacute intestinal obstruction, often leading to repeated conservative management before a definitive diagnosis is established [2-4].

We report a rare case of midgut volvulus secondary to intestinal malrotation in an 11-year-old child who presented with acute intestinal obstruction but without bowel ischemia. Early radiological diagnosis and timely surgical intervention resulted in complete bowel preservation and favorable postoperative recovery.

## Case presentation

This case was managed at a tertiary care teaching hospital in eastern India. An 11-year-old boy presented to the emergency department with sudden-onset bilious vomiting, abdominal pain, progressive abdominal distension, and obstipation for two days. He had experienced four previous episodes of subacute intestinal obstruction over the preceding three years, all of which were managed conservatively at peripheral healthcare facilities. The patient also had a history of an open appendectomy performed three years earlier.

On examination, the patient had a pulse rate of 108 beats/min, blood pressure of 104/68 mmHg, respiratory rate of 22 breaths/min, temperature of 37.1°C, and oxygen saturation of 98% on room air. Abdominal examination revealed generalized distension with mild diffuse tenderness and hyperactive bowel sounds. No signs of peritonitis were noted. Laboratory investigations revealed a hemoglobin of 12.8 g/dL, a total leukocyte count of 9.6 × 10⁹/L, a platelet count of 280 × 10⁹/L, a serum sodium of 133 mmol/L, a serum potassium of 3.6 mmol/L, a blood urea of 24 mg/dL, and a serum creatinine of 0.9 mg/dL.

**Figure 1:**
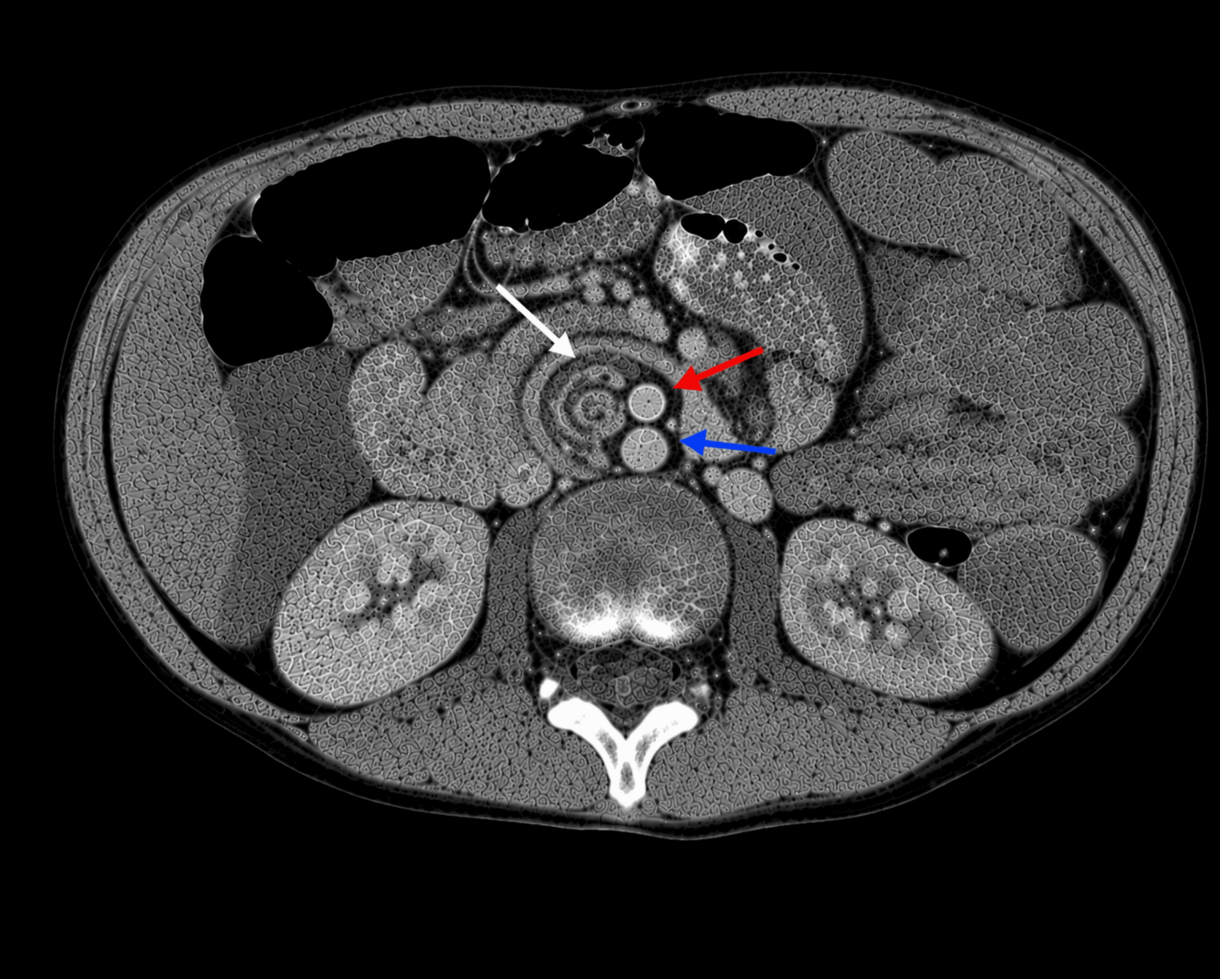
Contrast-enhanced computed tomography (CECT) scan of the abdomen demonstrating the classical whirlpool sign in midgut volvulus secondary to intestinal malrotation. Twisting of the mesenteric vessels around the superior mesenteric artery (SMA) is seen at the mesenteric root. The red arrow indicates the SMA, the blue arrow indicates the superior mesenteric vein (SMV), and the white arrow indicates twisting of the mesenteric vessels surrounding the mesenteric root.

Plain abdominal radiography demonstrated multiple dilated bowel loops suggestive of intestinal obstruction. Given the patient's hemodynamic stability, absence of peritoneal signs, and history of recurrent episodes of subacute intestinal obstruction, contrast-enhanced computed tomography (CT) of the abdomen was performed to identify the underlying cause of obstruction and assess bowel viability. Contrast-enhanced computed tomography (CECT) of the abdomen revealed the classical “whirlpool sign” caused by twisting of mesenteric vessels around the superior mesenteric artery (SMA), consistent with midgut volvulus secondary to intestinal malrotation (Figure 1). No radiological evidence of bowel ischemia, perforation, or pneumatosis intestinalis was identified.

The patient was resuscitated with intravenous fluids, nasogastric decompression, electrolyte correction, and broad-spectrum antibiotics. Emergency exploratory laparotomy was subsequently performed under general anesthesia.

**Figure 2:**
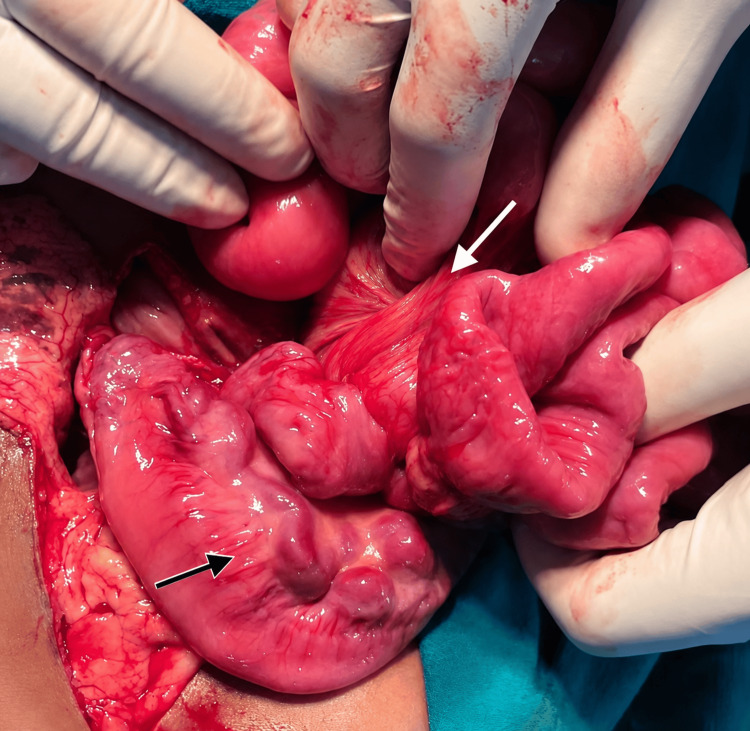
Intraoperative photograph demonstrating midgut volvulus with twisting at the root of the mesentery causing dilatation and congestion of the small-bowel loops. The white arrow indicates the twisted mesenteric root consistent with volvulus, while the black arrow indicates serosal venous congestion of the involved bowel loops.

Intraoperatively, a 180° clockwise midgut volvulus around a markedly narrow mesenteric base was identified (Figure 2). The bowel loops were congested but viable, with preserved mesenteric pulsations and improvement in color following derotation. No gangrene or transmural ischemic changes were present. Ladd's bands causing extrinsic compression were identified and divided. The duodenojejunal junction was located on the left side of the abdomen, while the caecum was situated on the right side but was excessively mobile. Taken together with the narrow mesenteric base and volvulus, these findings were considered consistent with incomplete intestinal malrotation.

A standard Ladd’s procedure was performed, including counterclockwise derotation of the volvulus, division of Ladd’s bands, widening of the mesenteric base, and repositioning of the bowel loops to reduce the risk of recurrent volvulus. Following derotation, bowel color improved significantly, mesenteric arterial pulsations were preserved, and normal peristalsis was observed. No transmural discoloration or serosal ischemic changes were present, confirming bowel viability. Because the caecum demonstrated marked mobility despite its right-sided location and the sigmoid colon was redundant, adjunctive caecopexy and sigmoidopexy were performed based on intraoperative judgment to reduce the risk of future torsion or recurrent obstructive episodes. We acknowledge that these fixation procedures are not routine components of Ladd's procedure and remain controversial. An appendectomy had already been performed previously; therefore, no further appendiceal procedure was required. No bowel resection was necessary.

Postoperatively, the patient was managed in the pediatric intensive care unit with continued nasogastric decompression, intravenous fluids, antibiotics, and gradual enteral feeding. Oral feeds were initiated on postoperative day 3 and tolerated well. The postoperative course remained uneventful, and the patient was discharged in stable condition on postoperative day 7.

## Discussion

Midgut volvulus is a potentially catastrophic complication of intestinal malrotation and represents a true surgical emergency. The condition predominantly presents during infancy, particularly within the first month of life, making delayed presentation in older children relatively uncommon [1-4]. Consequently, diagnosis beyond infancy is often difficult because symptoms may be intermittent, nonspecific, and recurrent over several years.

The present case is notable for several reasons. First, the patient presented at 11 years of age, which is unusual for symptomatic malrotation with volvulus. Second, the patient had experienced multiple prior episodes of subacute intestinal obstruction that were managed conservatively before a definitive diagnosis was established. Such intermittent symptoms may occur due to partial or transient volvulus and can delay diagnosis considerably [2].

Normal embryological development of the midgut involves physiological herniation followed by approximately 270° counterclockwise rotation around the SMA and subsequent fixation of the bowel to the posterior abdominal wall. Failure or interruption of this process results in a spectrum of intestinal rotational abnormalities, including non-rotation, incomplete malrotation, reversed rotation, and internal hernias. Complete non-rotation typically results in the small bowel lying predominantly on the right side of the abdomen and the colon on the left, whereas incomplete malrotation is characterized by variable abnormalities in rotation and fixation, including an abnormally positioned duodenojejunal junction, persistent Ladd's bands, excessive cecal mobility, and a narrowed mesenteric base that predisposes to midgut volvulus. In contrast, paraduodenal hernias arise from congenital mesenteric defects and represent the most common type of congenital internal hernia. Although both entities may present with recurrent intestinal obstruction, paraduodenal hernias do not result from the same mechanism of bowel fixation failure and are generally managed by reduction of the herniated bowel with closure of the mesenteric defect. Awareness of these embryological and anatomical differences facilitates accurate diagnosis and appropriate operative management.

Bilious vomiting remains one of the most important clinical warning signs of intestinal malrotation with volvulus in pediatric patients and should be considered a surgical emergency requiring urgent evaluation until proven otherwise, as delayed diagnosis may lead to catastrophic bowel ischemia and necrosis [5-6]. In older children, however, clinical presentation may mimic gastroenteritis, adhesive obstruction, functional abdominal pain, or other causes of recurrent vomiting, contributing to diagnostic delay. Atypical presentations of volvulus in older children and beyond infancy can pose a diagnostic challenge and may lead to delayed or missed diagnoses [5]. The patient had undergone an open appendectomy three years before presentation. Although operative records from the previous surgery were unavailable, recognition of abnormal bowel fixation, unusual caecal mobility, or atypical appendiceal location during that procedure might have prompted consideration of an underlying rotational anomaly.

Given the history of previous open appendectomy, postoperative adhesive intestinal obstruction was considered in the differential diagnosis. However, exploratory laparotomy did not demonstrate significant adhesions or adhesive bands contributing to obstruction. The obstructive episodes were instead attributable to midgut volvulus associated with a narrow mesenteric base and abnormal bowel fixation. This case highlights the importance of considering rotational anomalies even in patients with a history of prior abdominal surgery.

Although proximal intestinal obstruction due to malrotation with volvulus classically presents with a scaphoid or minimally distended abdomen in neonates, older children may demonstrate variable abdominal findings. Delayed presentation, prolonged obstruction, bowel edema, and accumulation of gas and fluid within dilated loops may result in abdominal distension, as observed in the present case.

Radiological imaging plays a crucial role in early diagnosis. Contrast-enhanced CT imaging can demonstrate characteristic findings such as the “whirlpool sign,” which represents the twisting of mesenteric vessels around the SMA. Early identification of this sign significantly facilitates prompt surgical management before the onset of irreversible bowel ischemia [7]. Although contrast-enhanced CT was diagnostic in the present case, other imaging modalities also play an important role in the evaluation of intestinal malrotation and midgut volvulus. Upper gastrointestinal contrast study remains the traditional reference standard for diagnosing malrotation by demonstrating abnormal positioning of the duodenojejunal junction and abnormal bowel rotation. Ultrasonography has increasingly been utilized as a rapid, non-invasive diagnostic tool and may demonstrate the characteristic whirlpool sign as well as abnormal SMA-SMV orientation. In patients presenting with acute intestinal obstruction, CT offers the additional advantage of evaluating bowel viability and identifying complications such as bowel ischemia, pneumatosis intestinalis, portal venous gas, or perforation. Therefore, the choice of imaging modality should be individualized according to clinical presentation, patient stability, and local expertise.

Ladd’s procedure remains the accepted standard surgical treatment for intestinal malrotation with midgut volvulus and aims to relieve volvulus, divide Ladd’s bands, broaden the mesenteric base, and place the bowel in a non-rotated configuration to minimize the risk of recurrent volvulus. Although recurrent volvulus and postoperative adhesive intestinal obstruction have been reported after Ladd’s procedure, these complications are uncommon, and most published studies have focused on identifying risk factors rather than recommending additional bowel fixation procedures. In contrast, the literature supporting adjunctive caecopexy or sigmoidopexy is sparse and consists predominantly of isolated case reports and small case series. Consequently, routine bowel fixation is not recommended in current pediatric surgical practice and remains controversial. In our patient, the presence of marked caecal hypermobility and a redundant sigmoid colon prompted selective fixation based on intraoperative judgment to reduce the theoretical risk of future torsion, recurrent volvulus, or postoperative internal rotation-related obstruction We acknowledge that the benefit of these adjunctive procedures has not been established by high-quality evidence and that further studies are required to define their role in the management of intestinal malrotation [8-10].

The present case reinforces that intestinal malrotation with midgut volvulus should remain an important differential diagnosis in any child presenting with bilious vomiting or acute intestinal obstruction regardless of age. Although delayed presentation beyond infancy is uncommon, failure to recognize this condition promptly may result in catastrophic bowel ischemia and intestinal loss. Once the diagnosis is suspected or established, early surgical intervention remains the cornerstone of management. The fundamental principles of Ladd’s procedure include counterclockwise derotation of the volvulus, complete division of Ladd’s bands, broadening of the mesenteric base, placement of the small bowel on the right and the colon on the left in a non-rotated configuration, and appendectomy when indicated. Adherence to these operative principles minimizes the risk of recurrent volvulus and preserves bowel viability. This case highlights that maintaining a high index of suspicion and performing timely surgical intervention are the key determinants of a favorable outcome.

In the present case, early diagnosis and timely surgical intervention prevented progression to bowel ischemia or necrosis. The bowel remained completely viable despite the presence of a volvulus, thereby avoiding bowel resection and its associated long-term complications, such as short bowel syndrome. Preservation of intestinal length significantly improves long-term nutritional and functional outcomes in pediatric patients.

This case emphasizes the importance of maintaining a high index of suspicion for intestinal malrotation and midgut volvulus even beyond infancy, particularly in children presenting with recurrent bilious vomiting or episodic intestinal obstruction. Early imaging and prompt operative management are essential for favorable outcomes.

## Conclusions

Midgut volvulus beyond infancy is rare and may present with non-specific or intermittent symptoms, resulting in delayed diagnosis. This case highlights the importance of considering intestinal malrotation with volvulus in older children presenting with recurrent episodes of bilious vomiting or subacute intestinal obstruction.

Prompt radiological evaluation, particularly identification of the “whirlpool sign” on CT, allows early diagnosis and timely surgical intervention before the onset of bowel ischemia. Adjunctive bowel fixation procedures such as caecopexy and sigmoidopexy may be beneficial in selected patients with excessive bowel mobility or recurrent obstructive symptoms. Early performance of Ladd’s procedure can preserve bowel viability, prevent catastrophic complications, and result in excellent postoperative outcomes.
